# Supplementary material for: Teletherapy and hospitalizations in patients with serious mental illness during the COVID-19 pandemic: A retrospective multicenter study
Source: PLoS One. 2022 Apr 18;17(4):e0267209. doi: 10.1371/journal.pone.0267209 (PMC9015154; doi:10.1371/journal.pone.0267209)
Supplement: S1 File — (PDF) [file pone.0267209.s001.pdf]

PROPORTION COMPARISON
(difference between two proportions)

		Test type
**Test type** (1.unilateral o 2.bilateral)	1	UNILATERAL
**Confidence level** (1-α)		95%
**Power**		80%
P_1 Proportion sample A		20%
P_2 Proportion sample B		30%
**SAMPLE SIZE (n)**		**231**

SAMPLE SIZE ADJUSTED TO LOSS

**Expected loss proportion** (R)	15%
**Sample adjusted to loss**	**272**

Beatriz López Calviño
Salvador Pita Fernández
Sonia Pértega Díaz
Teresa Seoane Pillado
Unidad de epidemiología clínica y bioestadística
Complexo Hospitalario Universitario A Coruña
