# Supplementary material for: Teletherapy and hospitalizations in patients with serious mental illness during the COVID-19 pandemic: A retrospective multicenter study
Source: PLoS One. 2022 Apr 18;17(4):e0267209. doi: 10.1371/journal.pone.0267209 (PMC9015154; doi:10.1371/journal.pone.0267209)
Supplement: S1 Checklist — (PDF) [file pone.0267209.s002.pdf]

STROBE Statement—Checklist of items that should be included in reports of ***cohort studies***

	Item No	Recommendation
**Title and abstract**	1	(*a*) Indicate the study's design with a commonly used term in the title or the abstract
		The title includes the study´s design (retrospective multicenter study)
		(*b*) Provide in the abstract an informative and balanced summary of what was done and what was found
		The abstract includes information on background, methods, results, and main conclusions
**Introduction**		
Background/rationale	2	Explain the scientific background and rationale for the investigation being reported
		Check pages 4 to 7 of the manuscript
Objectives	3	State specific objectives, including any prespecified hypotheses
		Check page 7 reporting the specific objectives of the study. We did not have any prespecified hypotheses, given that the study was exploratory focusing on a novel phenomenon (use of teletherapy during the COVID pandemic)
**Methods**		
Study design	4	Present key elements of study design early in the paper
		The study design is stated at page 8 of the manuscript
Setting	5	Describe the setting, locations, and relevant dates, including periods of recruitment, exposure, follow-up, and data collection
		The setting, study sites, data collection periods are included in the section Setting and Participants (page 8)
Participants	6	(*a*) Give the eligibility criteria, and the sources and methods of selection of participants. Describe methods of follow-up
		The sample eligibility, inclusion criteria and selection process is described at the section Setting and Participants (pages 8 and 9)
		(*b*) For matched studies, give matching criteria and number of exposed and unexposed
		Non applicable
Variables	7	Clearly define all outcomes, exposures, predictors, potential confounders, and effect modifiers. Give diagnostic criteria, if applicable
		Variable characteristics are described in the section Variables and Data sources/measurement (pages 9 and 10)
Data sources/ measurement	8*	For each variable of interest, give sources of data and details of methods of assessment (measurement). Describe comparability of assessment methods if there is more than one group
		Data sources are explained in the section Variables and Data sources/measurement (pages 9 and 10)
Bias	9	Describe any efforts to address potential sources of bias
Study size	10	Explain how the study size was arrived at
		The G power analysis is explained at the section Setting and Participants and is included as a Supplementary File (Check Sup. File 1)
Quantitative variables	11	Explain how quantitative variables were handled in the analyses. If applicable, describe which groupings were chosen and why
		The quantitative Variable in our study were age (in years), percentage of hospitalizations, and Mean (SD) visits at the Emergency Department. The variable

		measurement is explained at the Variables and Data sources/Measurement section.
Statistical methods	12	(*a*) Describe all statistical methods, including those used to control for confounding
		(*b*) Describe any methods used to examine subgroups and interactions
		(*c*) Explain how missing data were addressed
		(*d*) If applicable, explain how loss to follow-up was addressed
		(*e*) Describe any sensitivity analyses
		Statistical methods are described at the corresponding section (pages 11-12), addressing a and b. See Settings and Participants section for c. The d and e aspects are not applicable in this study
**Results**		
Participants	13*	(a) Report numbers of individuals at each stage of study—eg numbers potentially eligible, examined for eligibility, confirmed eligible, included in the study, completing follow-up, and analysed
		(b) Give reasons for non-participation at each stage
		(c) Consider use of a flow diagram
		The study was based on data retrieved from clinical records, therefore only participants with full data were included in the study. Check the Methods corresponding section (page 8)
Descriptive data	14*	(a) Give characteristics of study participants (eg demographic, clinical, social) and information on exposures and potential confounders
		(b) Indicate number of participants with missing data for each variable of interest
		(c) Summarise follow-up time (eg, average and total amount)
		Participants Characteristics can be found at page 12 and Table 1
Outcome data	15*	Report numbers of outcome events or summary measures over time
		Check percentages of each intervention modality used and differences between them (Table 2). Check percentages of hospitalizations, Mean and SD of Emergency Department visits and differences among intervention modalities (Table 3).
Main results	16	(*a*) Give unadjusted estimates and, if applicable, confounder-adjusted estimates and their precision (eg, 95% confidence interval). Make clear which confounders were adjusted for and why they were included
		Check unadjusted, confounder-adjusted estimates and precision at Table 4. Confounders justification can be found at the Discussion section (page 24)
		(*b*) Report category boundaries when continuous variables were categorized
		Not applicable
		(*c*) If relevant, consider translating estimates of relative risk into absolute risk for a meaningful time period
		Check Table 3
Other analyses	17	Report other analyses done—eg analyses of subgroups and interactions, and sensitivity analyses
		Not applicable
**Discussion**		
Key results	18	Summarise key results with reference to study objectives
		A summary of the findings based on the study objectives is provided at pages 19-21
Limitations	19	Discuss limitations of the study, taking into account sources of potential bias or imprecision. Discuss both direction and magnitude of any potential bias
		The study limitations including sources of bias through confounding variables are discussed at the section Limitations and Strengths, pages 22-23
Interpretation	20	Give a cautious overall interpretation of results considering objectives, limitations,

		multiplicity of analyses, results from similar studies, and other relevant evidence
		A cautious interpretation is provided at the section Key results and Interpretation (pages 19-21)
Generalisability	21	Discuss the generalisability (external validity) of the study results
		The generalisability of the results is discussed throughout the Discussion, to the extend were findings of a study carried out in the context of the COVID pandemic can be applied to the routine clinical practice
**Other information**		
Funding	22	Give the source of funding and the role of the funders for the present study and, if applicable, for the original study on which the present article is based
		Funding information is provided as requested

*Give information separately for exposed and unexposed groups.

Note: An Explanation and Elaboration article discusses each checklist item and gives methodological background and published examples of transparent reporting. The STROBE checklist is best used in conjunction with this article (freely available on the Web sites of PLoS Medicine at http://www.plosmedicine.org/, Annals of Internal Medicine at http://www.annals.org/, and Epidemiology at http://www.epidem.com/). Information on the STROBE Initiative is available at http://www.strobe-statement.org.
