# Supplementary material for: Teletherapy and hospitalizations in patients with serious mental illness during the COVID-19 pandemic: A retrospective multicenter study
Source: PLoS One. 2022 Apr 18;17(4):e0267209. doi: 10.1371/journal.pone.0267209 (PMC9015154; doi:10.1371/journal.pone.0267209)
Supplement: Attachment — Submitted filename: PLOS paper review.docx [file pone.0267209.s003.docx]

Thanks for giving me the opportunity to review this important paper. The paper covers an important and current topic of remotely delivered mental health services during COVID-19 pandemic. I have few comments which may need to be addressed by the authors.

1) I think it would be a good idea if the authors provide more details for how the ‘treatment adherence’ was measured in the methodology section.
2) I wonder if the authors can explore further how the % of hospitalization was calculated? Was it calculated against the individual’s history of admission or to all patients in the current sample.
3) I am not sure if McNamar test was the appropriate test to examine the changes over the three periods of time. This test examines each couple of periods individually, so the error may be amplified over the three comparisons. I think Friedman test would be more fitting here.
4) Similarly, table three demonstrates individual lines of analyses (chi square or t test for each line), which I find difficult to interpret. It could be more comprehensible to apply one chi square analysis for one table (3x2) and the same for t test, instead ANOVA test would be used.
5) It is not clear why authors included these specific factors in the hierarchal regression model (specific sociodemographic or clinical). I may suggest adding some details in the introduction section to justify the work.
6) From table 4, I would appreciate if the authors could provide the Wald value for the significant predictors to identify the strongest predictor, given that all interventions were significant, including the In-person intervention. Particularly when the CI of the Videoconference intervention is quite high.
