# Supplementary material for: Teletherapy and hospitalizations in patients with serious mental illness during the COVID-19 pandemic: A retrospective multicenter study
Source: PLoS One. 2022 Apr 18;17(4):e0267209. doi: 10.1371/journal.pone.0267209 (PMC9015154; doi:10.1371/journal.pone.0267209)
Supplement: Attachment — Submitted filename: Response to the Reviewers_1Apr22.docx [file pone.0267209.s005.docx]

**Manuscript ID PONE-D-21-24939-R1**

**Response to the Reviewers’ comments**

**Reviewer #1: I would like to thank the authors for the prompt reply to the raised points.

Only few concerns are still there:
1) Thanks for using Cochrane’s test, I think, therefore the authors would need to report McNemar’s test with manually Bonferroni correction, rather than McNemar’s test only.**

*_We added a sentence in the Statistical Methods section specifying the Bonferroni correction applied (page 11)*

*_ To reflect the applied correction to the results, we modified the corresponding Results section (page 14)*

**2) Table 3 seems quite confusing to me. The title refers to six months after the first wave, while in the table there are two months after the lock down. Similarly, in narration, the two terms were used interchangeably “Hospitalization rates two months after the first wave did not differ between patients who received …”, please consider revision**

*___We have now revised the Table title and the text to provide consistency*

**3) I may disagree with “… with slight risks associated with receiving in-person interventions (OR=0.56; p=.03)”, this seems to me not a risk, however in-person interventions seems going in the same direction as videoconferencing and interventions over the phone, with a lower risk for hospitalization.**

*___We have now modified this sentence to provide clarity, as follows: “These findings were maintained when including potential confounders, with lower risk for hospitalizations associated with receiving in-person interventions(…)”*

**4) A typo in “(p = .11NS)” and in Table 4 “-0,69 (0.23)”**

*__Thank you for the suggestion, these typos are corrected*

Reviewer #3: Peer review PONE

**Dear Authors,
Thank you for the thorough responses to my queries. I think a final point on the diagnosis question, although you have addressed this very thoroughly with the changes to the method and discussion, is maybe to hint at the fact that diagnoses in mental health are all collections of symptoms, or syndromes, and could be explained using completely different lenses to the medical lens. This, I think, would aid in making the argument for why it was justified, and better, that you did not stick very strictly to one country’s narrow diagnostic category, from a single point in time, but rather took a more inclusive view, that reflects the uncertainties around the nature and aetiology of mental illness and our ever-evolving understandings.
Some references to include in making this point (a sentence in the discussion or methods would suffice I believe):
Bhui, K., & Priebe, S. (2006). Assessing explanatory models for common mental disorders. The Journal of clinical psychiatry, 67(6), 1441.
Conneely, M., Higgs, P., & Moncrieff, J. (2021). Medicalising the moral: the case of depression as revealed in internet blogs. Social Theory & Health, 19(4), 380-398.**

*__We thank the Reviewer for the suggestion, we have now added a sentence at the Discussion section: See Limitations, p.22*

**There are a few minor changes that need correcting that should be picked up in a thorough proof-read, a few are picked up below:
1. Repetition of the word “challenging” in the concluding paragraph of the abstract.**

*__The word is now erased*
**2. “Less” should be “fewer” in the last line of the Results section of the abstract.**

*_”Less” is replaced with “fewer”*
**3. First paragraph of the discussion, last sentence, there is an unnecessary “of”**

*__”Of” is erased*
**4. Discussion: “crise” ◊ “crisis”**

*__This is now replaced*

*Once again, we thank the Reviewers for their valuable comments that brought considerable improvements to our manuscript*
